# Structure of a MenB de-N-acetyl polysialic acid antibody and mechanism of immune cell inhibition

**DOI:** 10.1016/j.jbc.2026.113249

**Published:** 2026-06-12

**Authors:** Gregory R. Moe, Jon Agirre, Peter T. Beernink

**Affiliations:** 1 Saccharo, Inc., San Francisco, California, USA; 2 Department of Chemistry, University of York, York, UK; 3 Department of Pediatrics, San Francisco School of Medicine, University of California, San Francisco, USA

**Keywords:** cytokines, de-N-acetyl sialic acid, *Neisseria* meningitidis serogroup B, NK cells, polySia, T cells

## Abstract

The *Neisseria meningitidis* serogroup B (MenB) polysaccharide, α(2–8) polysialic acid (polySia), is essential for resistance to complement-mediated bacteriolysis and inhibition of opsonophagocytosis. The monoclonal antibody SEAM 3 binds de-N-acetyl polySia (dPSA) but not polySia, yet still recognizes encapsulated MenB, indicating the capsule contains dPSA derivatives. Since SEAM 3 also recognizes other pathogens as well as human cancers, we have explored its biological role and mapped the SEAM 3 epitope. We compared binding of N-acetylated and de-N-acetylated polySia and ganglioside GD3 derivatives to sialic-acid-binding immunoglobulin-like lectins (Siglecs) on leukocytes. Siglec-9 showed higher affinity for dPSA and de-N-acetyl GD3 (K_D_ ∼2.6–3.1 nM) than for their acetylated counterparts, while Siglec-5 bound polySia and GD3 derivatives with similar and moderate (K_D_∼40–60 nM and 7.9 nM, respectively) affinity. Siglecs-2, -3, -7, -10, and -11 showed negligible binding. T cells, NK cells, and monocytes incubated with live dPSA-expressing MenB acquired dPSA and bacterial protein. A lipophilic dPSA derivative also suppressed endotoxin-induced secretion of IL-1β, IL-6, and TNFα from human peripheral blood mononuclear cells. To map the SEAM 3 epitope, we co-crystallized a humanized SEAM 3 Fab with a dPSA derivative, resolving the structure at 1.83 Å (Rfree = 0.230) with good geometry. The epitope comprises four residues, with a de-N-acetylated residue at the non-reducing end. These findings suggest that dPSA may help mediate MenB immune evasion by engaging the inhibitory Siglec receptors 5 and 9 and confirm the utility of SEAM 3 as a structural probe of dPSA biology and the role of Siglecs in MenB pathogenesis.

The capsular polysaccharide of *Neisseria meningitidis* serogroup B (MenB) bacteria, α(2–8) polysialic acid (MenB PS or polySia), is an essential virulence factor enabling survival of MenB bacteria in the human blood stream by inhibiting complement activation (1) and osonophagocytosis (2). The capsular polysaccharides are also important for resistance to anti-microbial peptides (3). Unlike the sialic acid-containing capsular polysaccharides of other meningococcal serogroups such as A, C, W, X, and Y the group B capsular polysaccharide is poorly immunogenic in humans (4). This lack of immunogenicity likely arises from its structural identity with polySia produced by humans during embryonic development (5) and in select tissues such as regenerative tissues in the brain and white blood cells post development (6).

To overcome immunological tolerance and develop a capsular polysaccharide-based vaccine to prevent disease caused by MenB, Jennings and coworkers removed the N-acetyl groups from MenB PS and re-acylated it with propionyl groups (NPr-MenB PS). Conjugation of NPr-MenB PS to tetanus toxoid, elicited antibodies that bound to MenB and mediated serum bactericidal activity (SBA) (7). SBA is a surrogate for protection against meningococcal disease. Importantly, a subset of anti-NPr-MenB PS monoclonal antibodies (mAbs) mediated SBA against MenB but did not cross-react with polySia (8). Our group produced a panel of mAbs using a similar NPr-MenB PS conjugate vaccine. Our vaccine replicated the results of Jennings and coworkers with respect to a subset of mAbs being reactive with both MenB bacteria and NPr-MenB PS but not polySia (9). Subsequently, we discovered several of the antibodies produced were specific for binding to de-N-acetyl polysialic acid (dPSA), which was a minor by-product in the vaccine preparation resulting from incomplete re-acylation. Unlike polySia, dPSA was immunogenic in mice (10). dPSA was not a derivative of polySia known to occur naturally in any species. However, de-N-acetyl polysaccharides and polysaccharide de-N-acetylases are present in other species (11) and other investigators have described de-N-acetyl sialic acid derivatives of gangliosides GM3 (12) and GD3 (13, 14) expressed by cancer cells. Remarkably, two monoclonal antibodies, SEAM 2 and SEAM 3, which were raised against NPr-MenB PS, bound to other microbial pathogens, such as *Leishmania major* (15), intracellular perinuclear antigens in some human tissues, a subset of normal peripheral blood mononuclear cells (PBMC), the surface of embryonic extravillous trophoblasts (16), and cells of many different human cancers (17, 18).

Humans express two polysialyltransferases, ST8SIA2 and ST8SIA4, that produce polySia that modifies a few proteins, primarily, neural cell adhesion molecule in humans (19). ST8SIA2 is expressed almost exclusively during fetal development (20) while ST8SIA4 expression is limited mainly to white blood cells post development (21). Either or both polysialyltransferases can be aberrantly expressed in cancer where they are associated with metastasis and poor prognosis (22). We found that binding of SEAM 2 or SEAM 3 to human SK-MEL-28 melanoma and CHP-134 neuroblastoma cells depended on expression of ST8SIA2 (17). The results suggested that the presence of dPSA on cancer cells may be linked to ST8SIA2 expression. Although SEAM 2 and SEAM 3 were highly specific for binding to dPSA by ELISA, the protein co-immunoprecipitated by the mAbs from cancer cells and human normal PBMC was nucleolin (17), not neural cell adhesion molecule or any other protein known to be modified with polySia. Nucleolin is a highly modified, multi-functional protein found in the nucleus and cytoplasm but is not normally on the surface of cells (23). Studies by other investigators have shown that nucleolin is sialylated and have provided evidence that it can be on the surface of cancer cells (24). Nucleolin, however, is not known to be polysialylated.

Since dPSA is a novel derivative of polySia, we investigated whether sialic binding lectin receptors (Siglecs) present on immune cells bind to dPSA and de-N-acetyl sialic acid-containing derivatives of ganglioside GD3. We found that Siglec-9, a Siglec that inhibits the function of immune cells that express it, binds to dPSA with high affinity. Further, dPSA expressed by MenB bacteria was transferred to immune cells, which may be a mechanism for MenB to suppress immune responses. These findings raise a key question: what is the molecular basis for recognition of de-N-acetyl polysialic acid? Here, we confirm the structure of the saccharide epitope bound by SEAM 3 by crystallizing a humanized variant of the SEAM 3 Fab in complex with a dPSA tetramer selected from the original vaccine preparation of N-Pr MenB PS used to produce SEAM 3 and determined the structure by X-ray crystallography.

## Results

### Fc-Siglec receptor binding to de-N-acetyl sialic acid derivatives

**Figure 1:**
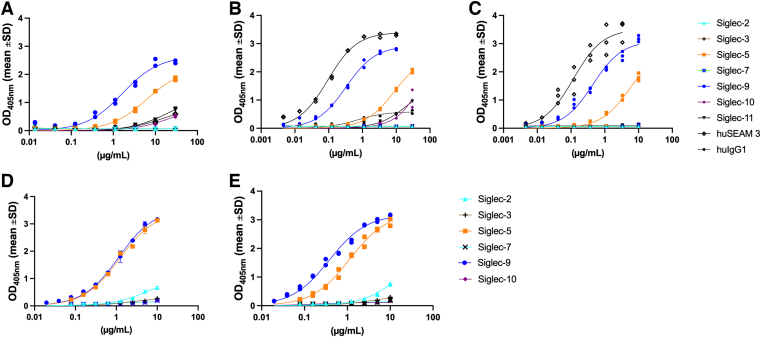
**Siglec receptor binding tosialic acid derivatives.** PolylSia (*A*), dPSA-ADH (*B*), dodecylamine-dPSA (*C*), GD3 (*D*) and dGD3 (*E*) by ELISA. The graphs shown are representative of experiments performed as described in experimental procedures in duplicate (*A*, *B*, *D*, *E*) or triplicate (*C*) technical replicates with the antigens adsorbed to the plate and the recombinant Fc-Siglec receptors or antibody added in solution. The binding data was fitted to a non-linear hyperbolic binding isotherm and binding constants calculated from the fitted curves using GrapPad Prism 10.6.1. The mean ± SD for K_D_s are provided in Table S1. dPSA, de-N-acetyl polySia.

Since dPSA has not been identified as a naturally occurring derivative of polySia other than the observed binding of SEAM 2 and SEAM 3 to dPSA on MenB bacteria and cancer cells (10, 17, 18), we investigated whether Siglec receptors were able to bind to dPSA containing 50% de-N-acetylated residues. Recombinant Siglec-2, -3, -5, -7, -9, -10 and -11 binding domains fused to human immunoglobulin Fc domains were used in direct binding ELISAs. Binding of the Fc-Siglecs to a polySia- adipic acid di-hydrazide (ADH) derivative was compared to binding to dPSA-ADH prepared the same as polySia-ADH and dodecylamine-dPSA in which dodecylamine is linked to the dPSA through C8 at the nonreducing end of the polymer (25). The ADH derivatives of polySia and dPSA and the dodecylamine-dPSA derivative contain a mixture of polysaccharides ranging from 2 to ∼30 residues as determined previously (9). The binding of the recombinant Siglecs to polySia-ADH, dPSA-ADH and dodecylamine-dPSA is shown in Figure 1, *A*–*C* respectively. There was no binding of Siglec-2, -3, -7, -10, or -11 to polySia-ADH, dPSA-ADH or dodecylamine-dPSA. Siglec-5 and -9 bound to polySia-ADH (Fig. 1*A*) with K_D_s of 43.6 nM and 13.2 nM, respectively. Siglec-10 and -11 and humanized SEAM 3 (huSEAM 3) had some background binding that was not different from the irrelevant human IgG1 negative control Ab. Siglec-9 binding to dPSA-ADH (K_D_ = 2.59 nM) was ∼5-fold greater than to polySia while Siglec-5 binding to dPSA-ADH (K_D_ = 61.1 nM) was comparable to its binding to polySia-ADH. The positive control Ab, huSEAM 3, had high affinity binding to dPSA-ADH (K_D_ = 0.64 nM) and dodecylamine-dPSA (K_D_ = 0.81 nM) and did not bind to polySia-ADH. The results for Siglecs-5 and -9 and SEAM 3 binding to dodecylamine-dPSA were approximately the same as binding to dPSA-ADH (both having Dp > 10), indicating that dPSA derivatives modified at the C1 carboxyl group or through the non-reducing end residue at C8 did not interfere with binding to the Siglec-5 and -9 or huSEAM 3.

The first description of sialic acid containing glycan with de-N-acetyl residues (dPSA) was described by Hani *et al.* for ganglioside GM3 (12) and Sjoberg *et al.* for ganglioside GD3 (26). Since GD3 contains a four-residue oligosaccharide with two α(2–8)-linked sialic acid residues at the non-reducing end is similar to the epitope recognized by huSEAM 3 in the X-ray structure described below with respect to length and having potential de-N-acetyl sialic acid at the nonreducing end, we compared Siglec and huSEAM 3 binding to GD3 and de-N-acetyl GD3 (dGD3). huSEAM 3 did not bind to either GD3 or dGD3. Siglecs-2, -3, -7, and -10 (Siglec-11 was not tested) did not bind to either GD3 or dGD3 (Fig. 1, *D* and *E*). Siglec-5 bound to GD3 and dGD3 (Fig. 1, *D* and *E*, respectively) with similar high affinity (K_D_ = ∼8 nM) and Siglec-9 bound to dGD3 (K_D_ = 3.02 nM) about 2-fold better than GD3 (K_D_ = 7.88 nM). Table S1 summarizes the binding data for each of the Siglecs to all derivatives.

### dPSA is transferred from MenB bacteria to T cells, monocytes and NK cells

**Figure 2:**
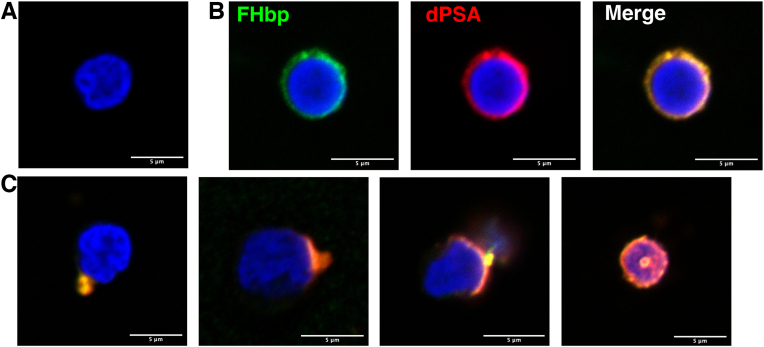
**Fluorescence microscopy of MenB strain H44/76 transferring dPSA to PBMC.***A*, PBMC in the absence of bacteria and stained with the same markers and secondary antibodies as in (*B*). *B*, fluorescence micrograph of a cell showing overlap of markers of outer membrane associated molecules including the bacterial lipoprotein FHbp (*green*) and capsular dPSA (*red*). The overlap for the two markers is *yellow* to *orange*. The cell nuclear DNA (*blue*) is stained with DAPI. *C*, four examples of bacteria at different stages of FHbp/dPSA transfer to cells at the same time point. The scale bar represents 5 μm in each of the micrographs. dPSA, de-N-acetyl polySia

Siglecs-5 and -9 contain an intracellular immunoreceptor tyrosine-based inhibitory motif (ITIM) domain and can be expressed by T cells, NK cells, monocytes and neutrophils (27). Ligand binding to Siglec-5 and -9 results in inhibiting the functional activity of cells expressing the receptor through the ITIM domain. To determine whether dPSA present in the capsular polysaccharide of MenB bacteria binds to white blood cells that express Siglecs-5 and -9, we incubated PBMC with MenB strain H44/76. The mixture of PBMC and bacteria were spotted onto glass coverslips for staining with JAR5 (IgG2a), a mAb that binds to meningococcal Factor H binding protein (FHbp) ID1, which is a lipoprotein, and SEAM 3 (IgG2b) to mark dPSA. Secondary antibodies conjugated to Alexa Fluor dyes conjugated to antibodies specific for mouse IgG2a (Alexa Fluor 488) and IgG2b (Alexa Fluor 596) subclasses were added to fluorescently label each of the antigens. The samples were analyzed by laser scanning confocal fluorescence microscopy. The micrographs in Figure 2*B* show labeling of a cell from a mixture of PBMC and bacteria where FHbp (green) and dPSA (red) cover the entire surface of the cell. As a control, PBMC stained with the same primary antibodies (*i.e.* JAR5 and SEAM 3), and Alexa Fluor-conjugated secondary antibodies in the absence of bacteria showed no fluorescent staining with anti-FHbp JAR5 or anti-dPSA SEAM 3 (Fig. 2*A*). In addition to the cell shown in Figure 2*B*, four individual cells from the mixture of PBMC and bacteria are shown in Figure 2*C* as examples of white blood cells and bacteria at different stages of interaction at the same time point. In the examples, it appears that FHbp and dPSA are transferred from the bacteria to the immune cells at the point of contact with the cells and subsequently appear to progressively migrate from the point of contact over the cell surface as in Figure 2, *B* and *C*.

**Figure 3:**
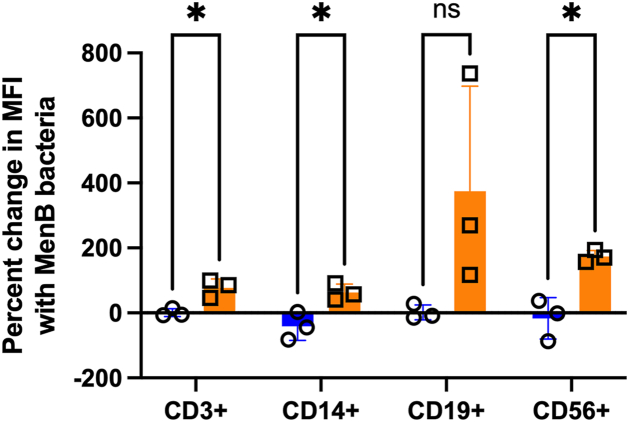
**Transfer of dPSA from MenB bacteria to PBMC.** Percent change in mean fluorescence intensity of anti-dPSA Ab SEAM 3 (*orange bars, square symbols*) or an irrelevant IgG2b control mAb (*blue bars, oval symbols*) binding to human PBMC in the absence *versus* presence of MenB strain 8047 were measured by flow cytometry using markers for T cells (CD3), monocytes (CD14), B cells (CD19) and NK cells (CD56). *Error bars* represent the standard deviation of the mean for three different donors with each transfer experiment performed independently on different days. Multiple unpaired *t* test statistical analysis and graphing were performed using GrapPad Prism 10.6.1. ∗, *p* ≤ 0.05; dPSA, de-N-acetyl polySia

To identify which PBMC acquire dPSA from MenB bacteria, the PBMC from 3 donors and MenB bacteria were combined in cell culture medium and incubated at ambient temperature for 30 min. MenB strain 8047 was used in this experiment because it is a high expressor of capsular polySia and dPSA. MenB capsular polysaccharide purified from MenB strains typically contains ∼50%-70% de-N-acetyl polySia as measured by modified resorcinol assay (28). After removing the bacteria by washing with cell culture medium and centrifugation, the PBMC were analyzed by flow cytometry using an irrelevant IgG2b negative control mAb, SEAM 3 to mark dPSA, and antibodies to CD3, CD14, CD19 and CD56 to mark T cells, monocytes, B cells and NK cells, respectively. Since PBMC express Fcγ receptors that can bind the Fc portion of the detecting mAbs and the expression of the Fcγ receptors can be modulated in response to the bacteria, we pre-blocked Fcγ receptors with Fc blocker and compared SEAM 3 and irrelevant IgG2b labeling in the absence and presence of MenB bacteria. The percent of cells of each cell type labeled with SEAM 3 was not significantly different either in the absence or presence of MenB bacteria. However, as shown in Figure 3, there was a significant increase of 63% to 174% (average of three donors) in SEAM 3-labeling of T cells, monocytes and NK cells as measured by the increase in the mean fluorescence intensity (MFI) of the cells after incubation with MenB but no change or a decrease in MFI of cells labeled with the irrelevant IgG2b control Ab. NK cells had a relatively larger increase in SEAM 3 binding (174% increase) than T cells or monocytes (Fig. 3). Larger changes in SEAM 3 MFI and increases in the percentage of labeled cells occurred with longer incubation time with the MenB bacteria but longer incubation times also resulted in a significant loss of PBMC viability. There was no change in PBMC viability (≥95%) with an incubation time of 30 min at ambient temperature.

### dPSA inhibition of inflammatory cytokine secretion by PBMC

**Figure 4:**
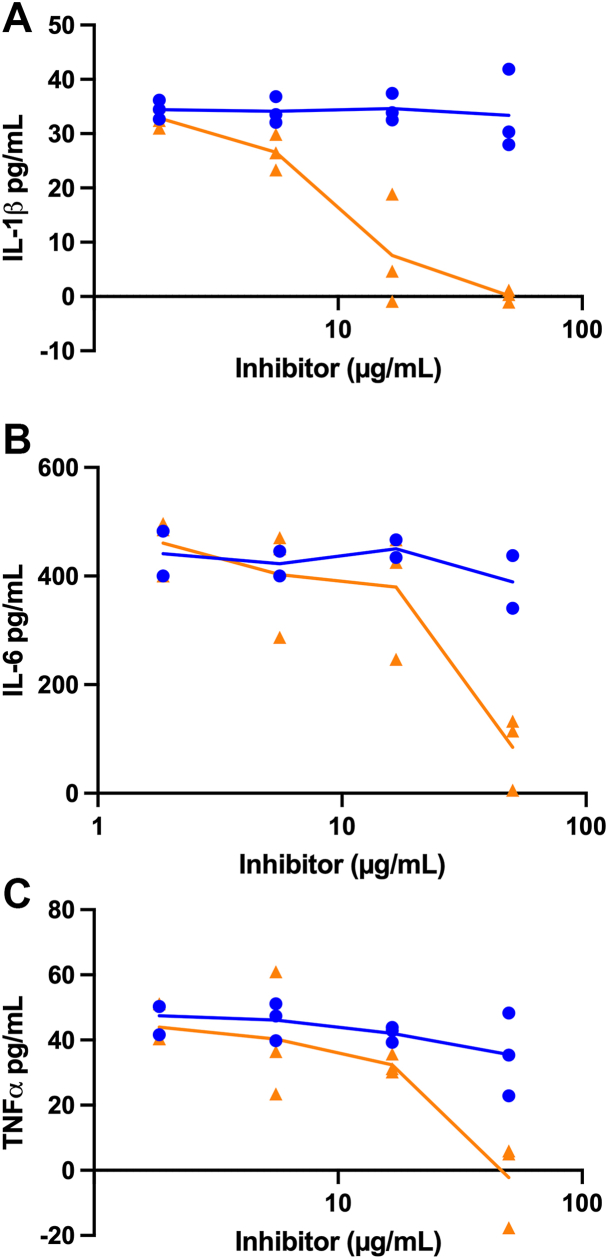
**Inhibition of PBMC cytokine secretion by de-N-acetyl sialic acid derivatives.** Effect dGD3 (*oval symbols, blue line*) and dodecylamine-dPSA (*triangle symbol, orange line*) on secretion of IL-1β (*A*), IL-6 (*B*) and TNFα (*C*) from PBMC stimulated with endotoxin in native outer membrane vesicles from MenB strain H44/76. Siglec-5 and Siglec-9 bind to dGD3 and dPSA (Fig. 1). In a separate experiment, soluble dPSA had no effect on cytokine secretion (data not shown). Each experiment was performed independently with PBMC from three different donors. Cytokine secretion in the absence of native outer membrane vesicles and presence of either dGD3 or dodecylamine-dPSA was less than the lower limit of quantitation. dPSA, de-N-acetyl polySia.

Siglecs-5 and -9 are inhibitory receptors. To determine whether dPSA derivatives have an inhibitory effect on the function of immune cells, we incubated PBMC with MenB native outer membrane vesicles (NOMV) to stimulate cytokine secretion in the absence and presence of the dodecylamine-dPSA, and dGD3 used in the ELISA binding assays. Dodecylamine-dPSA and dGD3 were chosen to test as inhibitors since both were bound by Siglecs-5 and -9 (Fig. 1, *C* and *E*) and can associate with cell membranes. As described in the experimental procedures, we were unable to synthesize a dodecylamine-polySia derivative without also generating dPSA epitopes. In a preliminary screening experiment, dPSA without dodecylamine modification and the polySia-ADH derivative had no effect on inhibiting cytokine secretion from PBMC but further work will be needed to confirm those results. PolySia and GD3 obtained from commercial sources were not tested since they contained endotoxin that resulted in cytokine secretion in the absence of NOMV (data not shown). Human PBMC from three different donors were treated with NOMV in the absence and presence of dodecylamine-dPSA or dGD3 for 18 h at 37 °C. Supernatants were collected from the wells and the concentrations of the inflammatory cytokines IL-1β, IL-6 and TNFα, one for each donor, were determined as described in the experimental procedures. Dodecylamine-dPSA inhibited secretion of the three cytokines but dGD3 had no effect (Fig. 4). The results show that dodecylamine-dPSA, which can associate with the cell membrane, may mimic the glycolipid capsular dPSA that was transferred from bacteria to the immune cells as shown in Figure 2, *B*, *C* and 3. However, dGD3, which can also associate with the cell membrane and bind to Siglecs-5 and -9 did not inhibit cytokine secretion (Fig. 4).

### Crystal structure of huSEAM 3 fab bound to dPSA

To define the structural basis for the interaction between huSEAM 3 and its dPSA ligand, a humanized variant of the huSEAM 3 Fab was co-crystallized with a derivative of NPr-MenB PS from the vaccine preparation used to elicit SEAM 3 in mice (9, 29). Attempts to cocrystallize the mouse SEAM 3 Fab with the same mixture of NPr-MenB PS oligosaccharides were unsuccessful although the mouse and chimeric mouse/human mAbs had identical binding to dPSA by ELISA. A comparison of the amino acid sequences of SEAM 3 and huSEAM 3 is shown in Fig. S2. We determined the structure of huSEAM 3 Fab bound to a NPr-MenB PS tetramer oligosaccharide by molecular replacement (see experimental procedures). The space group was P2_1_ and there were two copies of the Fab in the asymmetric unit of the crystal. We refined the structure at 1.83 Å resolutions to a working R factor (R-work) of 0.205 and a free R factor (R-free) of 0.237 with good geometry. The data collection and refinement statistics are listed in Table 1.

**Table 1:** Data collection and refinement statistics

Parameter	huSEAM 3 Fab-dPSA complex
Wavelength (Å)	1.116
Resolution range (Å)	74.49–1.86 (1.86–1.83)
Space group	P 1 21 1
Unit cell	74.585 71.437 91.495 90 92.95 90
Total reflections	579,953 (28,955)
Unique reflections	84,683 (4157)
Multiplicity	6.8 (7.0)
Completeness (%)	99.9 (99.9)
Mean I/sigma(I)	13.7 (0.8)
Wilson B-factor	33.6
R-merge	0.08
R-meas	0.234 (2.233)
R-pim	0.086 (0.841)
CC1/2	0.999 (0.406)
Reflections used in refinement	84,628 (8361)
Reflections used for R-free	1458 (138)
R-work	0.2048 (0.3412)
R-free	0.230 (0.3742)
Number of non-hydrogen atoms	7266
macromolecules	6599
ligands	259
solvent	408
Protein residues	865
RMS(bonds)	0.008
RMS(angles)	1.08
Ramachandran favored (%)	98.13
Ramachandran allowed (%)	1.87
Ramachandran outliers (%)	0.00
Rotamer outliers (%)	1.06
Clashscore	1.92
Average B-factor	38.65
macromolecules	37.75
ligands	47.43
solvent	47.66

Statistics for the highest-resolution shell are shown in parentheses (51).

**Figure 5:**
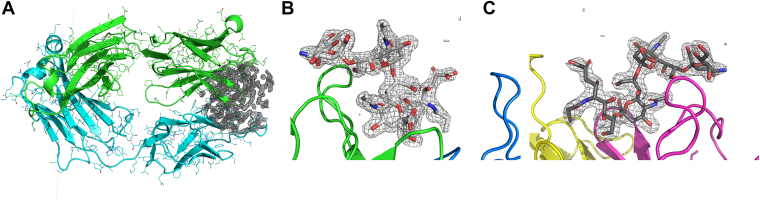
**Crystal structure of huSEAM 3 with dPSA oligosaccharide.***A,* overall structure of the huSEAM 3 Fab-dPSA tetramer structure (chains A, B, and E) in the asymmetric unit of the crystal structure. The heavy chain is shown in *green* and the light chain in *cyan*. The electron density map (2mFo-DFc, contour level 1 sigma) of the CDRs and bound sugar are shown for the huSEAM 3 Fab on the right of the figure. *B*, shake-omit electron density map for one copy of the dPSA tetramer (chain E). The heavy chain (chain A) is shown in *green* and the *light chain* (chain B) is shown in *blue*. *C,* shake-omit electron density map for one copy of dPSA tetramer (chain F). The heavy chain (chain C) is shown in *magenta* and the light chain (chain D) is shown in *yellow*. The atomic positions of Fab atoms were randomized to decrease model bias and the mFo-DFc map (contour level 3 sigma) was calculated without the dPSA tetramer atoms. CDR, complementarity determining region; dPSA, de-N-acetyl polySia.

The structure showed clear density for most of the Fab, including the complementarity determining region loops. One loop in the constant region of one copy of the light chain was disordered (chain D, residues 159–163). An overall view of the Fab structure is shown in Figure 5*A* along with an electron density map (2mFo-DFc, contour level 1 sigma) of the bound sugar for one of the Fabs (chains A, B and E). The two copies of the Fab showed nearly identical conformations (Fig. S3). The rms deviation between the two copies was 0.44 Å on all backbone atoms.

The bound dPSA tetramer included an open-chain form of an N-propionyl (N-Pr) residue 1 on the reducing end, followed by three residues in the pyranose form with two of the residues being the N-Pr derivative, and one residue of de-N-acetyl sialic acid at the non-reducing end. A shake-omit map showing electron density for the tetrasaccharide ligand is shown in Figure 5*B* (chains A, B, and E) and Figure 5*C* (chains C, D and F). The open chain conformation of the reducing end residue may have resulted from sodium borohydride reduction of the C2 ketone during the synthesis of the N-Pr derivative (29). However, the method used to produce N-Pr MenB PS with a defined degree of polymerization and conjugate it to a carrier protein in the vaccine used to elicit SEAM 3 (9, 29) would be expected to yield a reducing end sialic acid residue with a C2 keto group capable of adopting the pyranose structure. In addition, the N-Pr MenB PS antigens used to select the SEAM 3 hybridoma were made and purified the same as for the vaccine antigen but linked to biotin through carboxyl groups (9). Therefore, binding to the Fab combining site either stabilizes the open chain conformation or chains having a C2-OH at the reducing end residue were a minor contaminant in the vaccine preparation and elicited antibodies with specificity for the open chain conformation.

The conformations of the two copies of the polySia derivative were similar. While there was no electron density for the N-Pr group in the third residue of chain E, there was clear electron density for the N-Pr group in the third residue of chain F (B factor = 33.8 *versus* B = 42.7 for disordered residue). The environments of the third Sia residue were different between the two chains. In chain F, the third Sia residue was <4 Å from a symmetry-related molecule, which may have stabilized the N-Pr group in chain F. In contrast, in chain E the nearest symmetry mate was more distant (>9 Å) indicating that the N-Pr group of the third residue in this chain was not impacted by crystal contacts. Since it is unlikely that the bound tetra-saccharide has a different composition in the two copies of the asymmetric unit, the N-Pr group in chain E is more likely to be disordered than to be absent.

The N-propionyl derivative of polySia does not occur naturally, but SEAM 3 and huSEAM 3 bind to N-acetyl polySia dPSA derivatives with the same specificity but with lower affinity than N-propionyl dPSA derivatives (N-acetyl polySia dPSA tetramer K_D_ = 17 nM by inhibition ELISA (18) and 7.4 nM by SPR for Dp>10 N-acetyl polySia dPSA (Supporting information) vs 0.5 nM for N-propionyl polySia determined by inhibition ELISA (10)). The more hydrophobic propionyl group of residue 2 (18D 2), which is buried within the combining site may contribute to the increased affinity through hydrophobic interactions.

### huSEAM 3 Fab-polySia interactions

**Figure 6:**
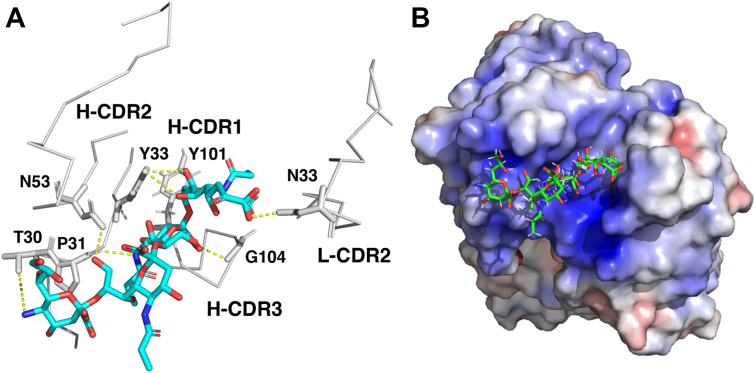
**Recognition of the dPSA oligosaccharide by huSEAM 3 Fab.***A*, Polar interactions between the huSEAM 3 Fab and the dPSA oligosaccharide. The four CDRs, three for the heavy chain (H1, H2, H3) and one (L2) for the light chain are shown in *gray* with the heavy chain CDRs indicated by H and the light chain CDR2 indicated by L. The dPSA tetramer carbon atoms are shown in *cyan*. huSEAM 3 Fab residues with direct bonding interactions with the dPSA oligosaccharide are as labeled with the interactions between atoms indicated by the *dotted yellow lines*. (*B*) Electrostatic surface rendering of the Fab with the dPSA tetramer in the combining site showing that, overall, the combining site is positively charged (charge range from +5 (*dark blue*) to −5 (*dark red*)). CDR, complementarity determining region; dPSA, de-N-acetyl polySia.

The tetra-saccharide ligand was bound in a groove formed primarily by the Fab heavy chain. The buried surface area was 1145 Å^2^. There were nine direct hydrogen bonding interactions between the Fab and the tetramer residues (Table 2). Of these, seven were mediated by the heavy chain and two mediated by the light chain. At the reducing end, Residue 1 (Y8W 1) formed 4 H bonds with the Fab, including two with the light chain. Residue 2 (18D 2) formed 4 H bonds with the heavy chain. Residue 3 (18D 3) formed 1 H bond with the heavy chain and residue 4 (Y98 4) did not have any direct hydrogen bonding interactions with the Fab (Fig. 6*A*; Table 2). However, the electron density clearly shows that residue 4 extends toward the surface of the binding groove. Combined with the observed specificity of SEAM 3 for polySia derivatives containing dPSA, this suggests that residue 4 plays an important role in binding. Specifically, the positively charged N5 amino group of residue 4 (Y98 4) likely forms stabilizing polar interactions with the negatively charged carboxyl group of residue 4, as well as with nearby hydroxyl group on Thr30 (complementarity determining region -H1). Together, these interactions help stabilize the conformation of residue 4 within the binding site. The C1-CO, C8-OH, C9-OH, and the heavy chain Thr30 hydroxyl are all within 4.8 Å to 5.5 Å of N5. The distances are within the range of significant charge-charge and charge-dipole interactions in proteins (30). In addition to the direct interactions between the Fab and the sugar, there are 8 bridging water molecules (Table S2*A*). Within the sugar, there were five direct intramolecular H bonds (Table S2*B*) as well as three mediated by water molecules (Table S2*B*).

**Table 2:** Direct bonding interactions between de-N-acetyl polySia and Fab

Chain	Residue (CDR)	Atom	dPSA residue	Atom	Distance (Å) Fab Ab/CD
Dipole-Dipole					
A/C	P31 (H1)	O	18D 2	N5	2.1/2.1
A/C	Y33 (H1)	OH	Y8W 1	O6	3.4/3.2
A/C	Y33 (H1)	OH	Y8W 1	O7	3.3/3.3
A/C	Y33 (H1)	N	18D 2	O4	2.9/2.0
A/C	N53 (H2)	ND2	Y98 3	O9	3.2/3.2
A/C	Y101 (H3)	O	18D 2	O4	2.6/2.6
A/C	G104 (H3)	N	18D 2	O1	1.9/2.0
B/D	N33 (L1)	ND2	Y8W 1	O2	3.5/5.5
B/D	N33 (L1)	ND2	Y8W 1	O1	3.4/2.5
Charge-Dipole					
A/C	T30 (H1)	OG1	Y98 4	N5	5.5/4.8

Several published studies suggested that the subset of antibodies elicited by NPr-MenB PS-based vaccines that bind MenB but not polySia recognize a helical epitope unique to the MenB capsule (31). The ϕ-ψ and ω_8_-ω_7_ backbone torsion angles of the oligosaccharide complexed with the huSEAM 3 Fab are not consistent with those predicted for a polySia helical structure (Table 3). Therefore, a helical structure appears to have no role in the epitope specificity of huSEAM 3.

**Table 3:** Backbone torsion angles for the de-N-acetyl polySia oligosaccharide^a^ in complex with the huSEAM 3 Fab

Angle	Y8W 1–18D 2^a^	18D 2–18D 3^a^	18D 3-Y98 4^a^	Helix (46)	PDB Sia average (46)
ϕ (°)	−65.8/−65.1	−63.3/−55.3	64.2/53.4	−45	−60/60
ψ (°)	135.0/132.2	121.9/116.0	114.2/110.8	130	∼120
ω_8_ (°)	61.8/61.4	66.5/57.7	−127.6/−118.6	60	
ω_7_ (°)	42.0/42.0	51.6/52.2	70.5/74.1	60	

a Values given for chains E and F, respectively.

## Discussion

The capsular polysaccharide is a critical virulence factor for pathogenic MenB, enabling the bacteria to evade multiple mechanisms of clearance including opsonophagocytosis, complement-dependent bacteriolysis and killing by antimicrobial peptides (1, 2, 3). The identification of antibodies produced using our preparation of the Jennings and co-workers’ N-Pr MenB PS vaccine (8) that bind with high specificity and affinity to polySia containing dPSA have led to the serendipitous discovery of dPSA (10, 15, 16, 17, 18, 28), a previously unrecognized derivative of polySia.

De-N-acetyl polysaccharides are produced by a variety of species (11). Moreover, de-N-acetyl sialic acid antigens such as de-N-acetyl gangliosides GM3 (12) and GD3 have been described previously (26). Our group has used SEAM 2 and SEAM 3 mAbs to identify dPSA in MenB (9, 10, 28), *L. major* promastigotes (15), and in several human cancers (16, 17, 18). The functional significance of the de-N-acetyl sialic acid derivatives has been unclear. However, Kasper and coworkers have demonstrated that de-N-acetyl variants of capsular polysaccharides made by other bacterial species (*e.g. Bacteroides fragilis*, *Streptococcus pneumoniae* type 1, and *Staphylococcus aureus* type 5 and type 8) elicit T-cell responses critical to immunological development and identified a possible pathway for their processing and presentation by MHCII to hypothetical carbohydrate-specific T cell receptors (32). Also, de-N-acetylated derivatives of poly-β-d-(1–6)-*N*-acetyl-glucosamine have been identified as key structural components of the biofilm extracellular polymeric substance of both Gram-positive and Gram-negative human pathogens (33). Like dPSA (28), de-N-acetylated derivatives of poly-β-d-(1–6)-*N*-acetyl-glucosamine is more immunogenic than the N-acetylated derivative. The zwitterionic chemistry of the derivatives (*i.e.,* polysaccharides that have both positive and negative charges) is important for processing and the observed functional activity. Although these examples are not directly related to dPSA, they provide evidence that polysaccharide de-N-acetylation is not unusual for microbial pathogens and that the human immune system has mechanisms for responding to it.

In this study, we have identified Siglecs-5 and -9 as receptors that bind to dPSA with nanomolar K_D_s. Siglecs-5 and -9 also bind to dGD3 with a nanomolar dissociation constants, suggesting Siglecs-5 and -9 could bind to a sialic acid-containing glycans that have as few as two sialic acid residues where one or both are de-N-acetylated. However, Siglec-5 binds equally to N-acetylated and de-N-acetylated derivatives of both glycans suggesting that de-N-acetylation is not important for binding to Siglec-5. In contrast, Siglec-9 shows some selectivity for de-N-acetylated derivatives with approximately 5-fold and 2-fold increased binding to dPSA and dGD3, respectively compared N-acetylated derivatives. The putative natural ligands for Siglec-5 are ΝeuAcα(2–8)-NeuAc or NeuAcα(2–6)GalNAc and α(2–3) or α(2–6) sialylated lactosamines such as sialyl-Lewis X for Siglec-9 based on glycan array studies (34). However, we found both Siglec-5 and -9 bound to α(2–8)-linked PSA/dPSA and GD3/dGD3 with an affinity 100- to 1000-fold greater than the putative natural or synthetic “high affinity” ligands, which have dissociation constants in the micromolar range (see, for example, (35)). One possible explanation for the difference is that we used fish gelatin as a blocking protein, which does not contain any contaminating sialic acid derivatives. For example, when we use bovine serum albumin as blocking protein in the ELISA assay, binding to polySia was reduced >10-fold while binding to dPSA derivatives were essentially the same as when using the fish gelatin or BSA as a blocking protein (Fig. S2). The standard protocol for blocking/dilution buffers used in glycan arrays often used for determining Siglec specificity, employ BSA (36), which may affect the results as described above.

Siglecs-5 and -9 are expressed on several types of white blood cells, including T cells, B cells, NK cells, monocytes and neutrophils (27) and are increased on tumor associated macrophages (37). The Siglecs inhibit the function of the cells through the cytoplasmic ITIM domain. Signaling can be dependent on receptor clustering that brings cytoplasmic domains in close proximity. Clustering facilitates recruitment of Src homology region domain-containing phosphatase-1 and 2, which dephosphorylate activated receptor proteins (38). Our findings demonstrate that dodecylamine-dPSA, which can associate with membranes, was able to inhibit secretion of inflammatory cytokines from PBMC. dGD3, which can also associate with membranes, did not affect cytokine secretion possibly because a higher concentration of single epitope dGD3 may have been needed *versus* dodecylamine-dPSA, which contains multiple two residue epitopes that appear to be recognized by the Siglecs, on a single chain. Additionally, we found that dPSA and a lipoprotein from MenB, FHbp, were transferred from the bacteria to PBMC. *N. meningitidis* and *Neisseria gonorrhoeae* are both known to bind to immune cells and transfer surface proteins such as porins (39). There are multiple ligands on MenB that mediate interactions with receptors on human cells including type IV pili, Opa/Opc proteins, and many others (reviewed in reference (40)). Possibly, adhesion of the bacteria to the immune cells is made through these other proteins with transfer of membrane components occurring after adhesion of the bacteria to the immune cells. The results from fluorescence microscopy and flow cytometry show that lipoproteins such as FHbp and glycolipid-linked dPSA polysaccharide are also transferred from the bacteria to immune cells. The ability of dodecylamine-dPSA to inhibit cytokine secretion suggests that signaling through the Siglecs may require a threshold density of de-N-acetylated sialic acid ligand on the surface of the cell to promote Siglec-9 clustering. Further studies will be needed to confirm a role for dPSA derivatives modulating immune cells through Siglec receptors.

Although prior studies demonstrated that mAbs SEAM 2 and SEAM 3 bind specifically to synthetic dPSA derivatives (10, 15, 16, 17, 18, 28), the X-ray structure of the huSEAM 3-dPSA complex provides key insight into the SEAM 3 epitope. The structure confirms that SEAM 3 recognizes a tetrameric polySia oligosaccharide with a dPSA at the nonreducing end (Figs. 5 and 6), consistent with previous epitope mapping using synthetic derivatives.

Only a small number of high-resolution three-dimensional structures exist for antibodies bound to sialic acid-containing glycans-roughly 5 to 15, depending on how scientific databases are searched. This scarcity is largely because sialic acid-containing glycans are inherently floppy and variable in shape, making them difficult to capture in a crystal structure. These same properties also interfere with crystal formation itself, so most antibody crystal structures either show no sugar chain detail at all, or only partial information with sialic acids, which sit at the ends of sugar chains, being especially prone to appearing blurred or absent.

Earlier work using computationally modeled antibody structures suggested that antibodies recognizing polySia tend to share certain structural features: they are encoded by a narrow set of germline antibody genes with few mutations, and their antigen-binding regions form wide, shallow, positively charged grooves (41). This shape is well-suited for binding an extended, repeating sugar chain. This description fits both the crystal structure of scFv735 and another anti-polySia antibody called 13D9 (42). Antibodies that instead recognize dPSA look quite different. Their combining sites contain pockets and deep clefts, which are better suited for recognizing specific structural features of individual residues (41).

Crystal structures of antibody–glycan complexes point to two broad ways that antibodies can bind sialic acid-containing glycans. First is direct contact: The antibody's binding loops make hydrogen bonds directly with the sugar. A clear example is the antibody LpMab-3 binding to a sugar chain on a protein called podoplanin. In this example, two sialic acid units make direct contact with four of the antibody's binding loops (H1, H2, H3, L3). The huSEAM 3 Fab binding to dPSA works similarly, with direct contacts formed between four binding loops (H1, H2, H3, L2) and the ligand (Fig. 6*A*). The second is water-mediated contact: Rather than contacting the ligand directly, the antibody uses a network of water molecules bridging the gap between protein and ligand. The best-characterized example involves scFv735 (derived from mAb735) bound to a chain of eight sialic acid units (43). Notably, the scFv735 complex is the only published crystal structure of an antibody complex with a polySia chain containing four or more residues besides the present study. In the scFv735 complex structure, 11 precisely positioned water molecules span the space between the antibody and the sugar chain, while direct antibody/glycan contacts are minimal. This water bridge is thought to compensate for a poor geometric fit between the flat binding surface of the antibody and the extended shape of the polySia chain. Interestingly, the eight-sugar chain is shared across two linked scFv735 molecules, and each binding site actually contacts only three of the eight residues, suggesting that longer polySia chains are needed not because each site binds more residues, but because having two binding sites working together (avidity) increases the overall strength of binding (43).

Despite using different binding strategies, the huSEAM 3 Fab and scFv735 share some features, both interact with only a small number of residues (four or fewer), both binding sites carry a net positive charge, and in both cases, tyrosine residues in the heavy chain make most of the direct contacts with the sugar's hydroxyl groups (Table 2). However, there are important differences. In the huSEAM 3 Fab/dPSA tetramer complex, the surface area buried at the Fab-dPSA interface is nearly three times larger (1145 Å^2^ for huSEAM 3 Fab *versus* ∼400 Å^2^ for scFV735). SEAM 3 also binds the entire glycan within a single binding site, whereas scFv735 distributes the octamer contacts across two linked binding domains. Additionally, one of the sugar units in the huSEAM 3 Fab complex adopts an unusual open-chain form. Finally, rather than bridging between the antibody and ligand, the few ordered water molecules in the huSEAM 3 Fab complex sit on the outer surface of the glycan ligand. Together, these structural differences, more extensive direct contact and a larger buried surface, likely explain why SEAM 3 binds dPSA with higher affinity than mAb735 and related antibodies that bind polySia.

In conclusion, these studies confirm the specificity of SEAM 3, which has been used to identify dPSA on microbial pathogens, human trophoblasts, and cancer cells and to highlight the possible role of dPSA-containing MenB capsular polysaccharides in immune invasion by suppressing immune cell function through Siglec-9 interactions. The data suggest that glycans containing de-N-acetyl sialic acid may have a broader role in immune modulation than previously known. Further studies will be needed to investigate whether specific de-N-acetylases target sialylated glycans, as these enzymes and de-N-acetylated glycans could serve as therapeutic targets for vaccines and inhibitors to combat diseases caused by microbial pathogens. Finally, with the X-ray structure of the SEAM3/dPSA tetramer complex and identification of Siglec-9 as a selective, high affinity binder of dPSA, SEAM 3 serves as a structural probe for defining the dPSA epitope relevant to Siglec-9 recognition.

## Experimental procedures

### Preparation of polySia and de-N-acetyl derivatives of polySia and GD3 used for ELISA

PolySia and dPSA were randomly modified at C1 carboxyl groups with ADH to facilitate coating on an ELISA plate. The ADH derivatives of colominic acid (Millipore Sigma) and dPSA were prepared as described previously (44). Partially de-N-acetylated polySia (*i.e.* dPSA) was prepared by combining colominic acid (100 mg) and 10 mg of sodium borohydride (Millipore Sigma) in 10 ml of 2M NaOH and heated to 90 °C in a sealed hydrolysis tube (Pierce Chemical Co.) for 40 min. After cooling the solution to ambient temperature, the solution was adjusted to pH 8.0 with 2 M HCl, dialyzed against water, and lyophilized. The preparation of dPSA contained 50% of de-N-acetyl residues as measured by modified resorcinol assay (28) that are randomly distributed throughout the polymer. The chemical procedures used to produce the polySia derivatives results in the fragmentation such that the preparation contains a mixture of polymer ranging in size from approximately 1 kDa to 30 kDa with the majority being <10 kDa as measured by anion exchange chromatography (Q Sepharose Fast Flow (Cytiva) as described previously (9).

Since modification of carboxyl groups with ADH, as described above, may affect binding of mAbs and Siglecs we also prepared a derivative of dPSA for ELISA containing dodecylamine at the non-reducing end (25). A similar derivative was not prepared for polySia since the procedure for making the dodecylamine derivative produces dPSA. To make dodecylamine-dPSA, an aldehyde group was introduced into the non-reducing end by dissolving 20 mg of dPSA in 1 ml of 0.1 M NaOAc buffer, pH 6.5. Sodium meta periodate (5 mg, Millipore Sigma) was added and the solution kept in the dark for 30 min. The remaining NaIO_4_ was degraded by adding 0.1 ml of 10% (wt/vol) ethylene glycol in water and left for 30 min. This procedure produces an aldehyde at C8 of the nonreducing end terminal residue. Dodecylamine-dPSA was prepared by combining 20 mg of dPSA containing a terminal end aldehyde with 10 mg of dodecylamine (Thermo Fisher Scientific) in 5 ml of water. After heating the mixture to 50 degrees C for 30 min with stirring, 5 mg of sodium cyanoborohydride was added. The mixture was stirred at ambient temperature for 24 h then dialyzed in 4L of water 3 times to remove excess dodecylamine. It was not possible to make a similar dodecylamine derivative of polySia since the high pH and high temperature used during reductive amination produced dPSA epitopes that bound to SEAM 3.

De-N-acetyl GD3 was prepared as described by Sonnenburg *et al.* (45) by suspending 1 mg of GD3 (Calbiochem) in 2 ml of 2.5 M tetrabutylammonium hydroxide and 3 ml of butanol then heating the mixture to 100 degrees C for 4 h. After cooling the mixture to ambient temperature, the pH was adjusted to about 7 by adding 2 M HCl and the butanol removed by evaporation under a stream of N_2_. The remaining aqueous layer was dialyzed (1K molecular weight cut-off dialysis tubing, Thermo Fisher Scientific) against PBS buffer and used without further modification as an antigen for ELISA.

### ELISA

The reactivity of recombinant Siglec-2, -3, -5, -7, 9, -10, -11-Fc receptors (R&D Systems) and huSEAM 3 with polySia-ADH, dPSA-ADH, dodecylamine-dPSA, GD3 or dGD3 was determined by direct binding ELISA in which the derivatives were adsorbed directly to the surface of a microtiter plate (Nunc MaxiSorp, Thermo Fisher Scientific) by incubating a solution of the antigen (5 μg/ml) in PBS buffer in the wells of the plate overnight at 4 °C. The plates were washed with PBS buffer (3×) and blocked with PBS buffer containing 0.1% (weight/volume) of gelatin from cold water fish skin and 0.1% Tween-20 (Millipore Sigma; blocking buffer) for 1 h at ambient temperature. Fish gelatin was used for blocking to eliminate any background of Siglecs binding to N-glycoyl Sia derivatives that might be present in blocking buffers containing proteins such as bovine serum albumin. The recombinant Siglec-Fc derivatives or antibodies were diluted in blocking buffer and added to the plate (50 μL per well) in duplicate. After incubating the plate for 1 h at ambient temperature, the plates were washed with PBS buffer (3×) and goat anti-human-alkaline phosphatase conjugated antibody (Jackson Immuno Research) diluted in blocking buffer was added. After incubating an additional 30 min, the plates were washed (3 times) with PBS buffer and the bound antibody was detected by adding p-nitrophenyl phosphate substrate in 50 mM sodium carbonate buffer, pH 9, containing 1 mM MgCl_2_. The absorbance at 405 nm after 20 to 30 min incubation at ambient temperature was measured using a Spectramax 340 (28) (Molecular Devices) or Bio-Tek Synergy H1 (Agilent Technologies) microtiter plate reader. The concentration dependence of the absorbance at 405 nm was fitted to a hyperbolic binding curve using GraphPad Prism 10.6.1 (https://www.graphpad.com/). Each binding experiment with polySia and dPSA derivatives were repeated at least three times and experiments with GD3 and dGD3 were repeated twice on different days.

### Laser scanning confocal fluorescence microscopy

MenB strain H44/76 was grown in chemically defined medium as described in Moe *et al.* (46). Human PBMC (Allcells) and bacteria were each washed in RPMI 1640 medium supplemented with 10% fetal bovine serum (Thermo Fisher Scientific). PBMC and bacteria were combined (1:10 ratio) in medium at ambient temperature for 30 min. The cells were collected by centrifugation (720×*g*), washed 2 times with medium, and aliquots were added to coverslips that had been treated with poly-L-lysine (Thermo Fisher Scientific). The cover slips were placed in a 24 well cell culture plate (Thermo Fisher Scientific) then fixed with 4% paraformaldehyde (Millipore-Sigma) in PBS at 4 °C for 1 h. The coverslips were washed with PBS and blocked overnight at 4 °C in PBS containing 4% goat serum (Millipore-Sigma) and 0.01%Tween-20. Primary antibodies (anti-dPSA mAb SEAM 3 (IgG2b) and anti-meningococcal Factor H binding protein mAb JAR5 (IgG2a)) were added in fresh blocking buffer and incubated at ambient temperature for 1 h. After washing 5 times with PBS buffer, AlexaFluor-488, and -594 -conjugated to goat anti-mouse IgG2a and IgG2b (InvitroGen), respectively were added in blocking buffer and incubated at ambient temperature for 1 h. The coverslips were washed with PBS 5 times and NucBlue DNA stain (InvitroGen) was added in PBS buffer. The coverslips were washed 2 times with water and mounted on glass slides. Micrographs were recorded using a Zeiss 710 (https://www.zeiss.com/microscopy/us/products/light-microscopes/confocal-microscopes.html) laser scanning confocal microscope.

### Flow cytometry

MenB strain 8047 was grown in Frantz medium supplemented with 2.5 mM lactate to an OD_620nm_ of 0.5 to 0.6. The bacteria were washed in RPMI 1640 medium (Thermo Fisher Scientific) containing 10% fetal bovine serum (cell culture medium). Cryopreserved human PBMC from three different donors were thawed and prepared for assay as recommended by the manufacturer (Allcells). PBMC were washed with cell culture medium then suspended in azide-free Fc Receptor Blocker (Innovex Biosciences) for 30 min at ambient temperature. The PBMC were collected by centrifugation as above and suspended in cell culture medium. The PBMC were divided into two aliquots and MenB strain 8047 diluted in cell culture medium was added to one aliquot of Fc receptor-blocked PBMC in a ratio of ∼10 to 50 bacteria to 1 PBMC. Fc receptor-blocked PBMC alone or with MenB bacteria were incubated at ambient temperature for 30 min. The PBMC were separated from the bacteria by centrifugation. An irrelevant IgG2b mAb (BioXCell) control or SEAM 3 (IgG2b) for detecting dPSA was added to aliquots of the cells. After 30 min at ambient temperature, the PBMC were collected as above and goat anti-mouse IgG2b-Alexa Fluor 488 (Invitrogen) and, individually, mouse anti-human CD3 (clone SK7), CD14 (clone M5E2), CD19 (clone HIB19), and CD56 (clone 5.1H11) antibodies conjugated to APC (all from BioLegend) were incubated with the PBMC at ambient temperature for 30 min. The PBMC were collected by centrifugation as above then fixed with 0.5% formaldehyde in PBS for 10 min and analyzed by flow cytometry on a NovoCyte flow cytometer (Agilent Technologies). The viability of PBMC without and with MenB bacteria were tested with Trypan Blue (Thermo Fisher Scientific) staining and counted on a hemocytometer (Incyto C-Chip, Thermo Fisher Scientific). Anti-Siglec-9 antibody (R&D Systems, clone 191240) and APC-conjugated anti-CD3, -14, -19, -56 mAbs were used to identify subpopulations of PBMC that express Siglec-9.

### Cytokine secretion from PBMC

Cryopreserved human PBMC from 3 donors were thawed and prepared for assay as recommended by the manufacturer (AllCells). The PBMC were suspended in RPMI 1640 medium supplemented with 10% fetal calf serum (Gibco) were added to the wells of a flat well microtiter plate (Thermo Fisher Scientific) containing a final concentration of 0.01 μg/ml of MenB strain H44/76 native outer membrane vesicles (NOMV) prepared as described in (47) with no derivative or serial dilutions of dodecylamine-dPSA, or dGD3. Ideally, dodecylamine-polySia would also have been used as a control in addition to dGD3 but we were not successful in making that derivative without generating dPSA epitopes as described above. The plates were incubated at 37 °C in 5% CO_2_ for 18 h. The plates were placed on ice and aliquots were added to wells in duplicate of test plates for IL-1β, IL-6, and TNFα provided in kits for each cytokine (Thermo Fisher Scientific). The cytokines were selected for study based on testing PBMC for cytokine secretion simulated with MenB NOMV on a cytokine array. The plates, including standards provided in the kits, were processed as recommended by the manufacturer and the concentrations of cytokine were determined from a standard curve. Each experiment was repeated with a different donor and cytokine on different days.

### Fab production and complex formation

The recombinant mouse SEAM 3 and humanized SEAM 3 (huSEAM 3) mAbs were produced in CHO cells and purified by Protein A/G affinity chromatography by WuXi Biologics. The mAbs were >98% pure by SDS-PAGE and analytical size-exclusion chromatography (SEC). The Fab fragment of huSEAM 3 was prepared by digestion with papain following the instructions provided in the Pierce Fab Preparation kit (Thermo Fisher Scientific). The huSEAM 3 Fab-dPSA complex was prepared by combining a mixture of N-Pr MenB PS oligosaccharides (Dp 3–10), prepared as described previously, with the purified Fab in a molar ratio of N-Pr MenB PS to Fab of 10:1. The N-Pr MenB PS preparation contained 16% dPSAs by NMR (9). The complex was purified by SEC (ToyoPerl HW 55F, Thermo Fisher Scientific) in 10 mM Tris, pH 7.5, 50 mM sodium chloride using an Äkta Explorer FPLC chromatography system (GE Healthcare Bioscience). The protein concentration was measured by UV spectroscopy (NanoDrop 1000, Thermo Fisher Scientific) using the extinction coefficient of the Fab calculated with ProtParam.

### Crystallization, data collection and analysis

Crystallization conditions were screened using sparse matrix kits (Hampton Research) in sitting drops. Crystals of the Fab-dPSA complex were optimized in hanging drops from an equal mixture of 5 mg/ml Fab/dPSA complex and precipitant solution containing 24% (w/v) polyethylene glycol 1500 and 20% (w/v) glycerol (optimization of CrystalScreen Cryo #43, Hampton Research). We collected single wavelength X-ray data (lambda = 1.116 Å) on beam line 8.3.1 at the Advanced Light Source, Lawrence Berkeley National Laboratory. We used the humanized huSEAM 3 Fab sequence to identify a search model for molecular replacement using Swiss-Model (https://swissmodel.expasy.org) (48), which identified a human-mouse chimeric Fab (PDB ID 6PE7) (49) as a suitable candidate. The structure was solved by molecular replacement (Phenix-MR; (50)); the crystals were in space group P2_1_ and there were two copies of the Fab/NPr-MenB PS complex in the asymmetric unit. The structure was refined (without noncrystallographic symmetry restraints) using iterative refinement with Phenix.refine (51) and manual model building with Coot (52) until the free R factor converged. We validated the structure with MolProbity (53) and analyzed the crystallographic data with PyMol (The PyMOL Molecular Graphics System, Version 2.0 Schrödinger, LLC). Carbohydrate residues were validated using Privateer (54). The coordinates are available in the Protein Data Bank with accession code 8FPA (http://www.rcsb.org/structure/8fpa).

## Data availability

The original microscopy images are stored on an external hard drive (Saccharo1) at Saccharo, Inc. and are available from G.R.M. The crystallographic data and PDB file are available at (http://www.rcsb.org/structure/8fpa). All other data and materials are contained completely within the article or supporting information. All data will be made fully available to any qualified investigator upon request.

## Supporting information

This article contains supporting information.

## Conflict of interest

The authors declare that they have no conflicts of interest with the contents of this article.
