# Supplementary material for: Structure of a MenB de-N-acetyl polysialic acid antibody and mechanism of immune cell inhibition
Source: J Biol Chem. 2026 Jun 12;302(8):113249. doi: 10.1016/j.jbc.2026.113249 (PMC13351141; doi:10.1016/j.jbc.2026.113249)
Supplement: Supporting Tables and Figures [file mmc1.docx]

**TABLE S1.** Summary of binding data for Siglecs and antibody controls binding to polySia, dPSA, dodecyl-dPSA, GD3 and dGD3 by ELISA.

|  | **Siglec-2** | **Siglec-3** | **Siglec-5** | **Siglec-7** | **Siglec-9** | **Siglec-10** | **Siglec-11** | **huSEAM 3** | **huIgG1** |
| --- | --- | --- | --- | --- | --- | --- | --- | --- | --- |
| **polySia-ADH** |  |  |  |  |  |  |  |  |  |
| Bmax (OD_405nm_) | ND^a^ | ND^a^ | 2.245 | ND^a^ | 2.651 | ND^a^ | ND^a^ | ND^a^ | ND^a^ |
| K_D_ (M) | ND^a^ | ND^a^ | 4.36E-08 | ND^a^ | 1.32E-08 | ND^a^ | ND^a^ | ND^a^ | ND^a^ |
| R squared | ND^a^ | ND^a^ | 0.9936 | ND^a^ | 0.9756 | ND^a^ | ND^a^ | ND^a^ | ND^a^ |
| Mean K_D_±SD for 3 assays | ND^a^ | ND^a^ | 4.46±0.24E-08 | ND^a^ | 1.44±0.24 E-08 | ND^a^ | ND^a^ | ND^a^ | ND^a^ |
| **dPSA-ADH** |  |  |  |  |  |  |  |  |  |
| Bmax (OD_405nm_) | ND^a^ | ND^a^ | 2.291 | ND^a^ | 3.465 | ND^a^ | 1.64 | 3.4 | ND^a^ |
| K_D_ (M) | ND^a^ | ND^a^ | 6.11E-08 | ND^a^ | 2.59E-09 | ND^a^ | 4.32E-08 | 6.40E-10 | ND^a^ |
| R squared | ND^a^ | ND^a^ | 0.9523 | ND^a^ | 0.9751 | ND^a^ | 0.961 | 0.988 | ND^a^ |
| Mean K_D_±SD (M) for 3 assays | ND^a^ | ND^a^ | 4.46±0.24E-08 | ND^a^ | 2.3±0.15 E-09 | ND^a^ | ND^a^ | ND^a^ | ND^a^ |
| **Dodecylamine-dPSA** | | | | | | | | | |
| Bmax | ND^a^ | ND^a^ | 2.862 | NA | 3.099 | ND^a^ | ND^a^ | 3.512 | ND^a^ |
| K_D_ (M) | ND^a^ | ND^a^ | 3.89E-08 | NA | 3.53E-09 | ND^a^ | ND^a^ | 0.81E-10 | ND^a^ |
| R squared | ND^a^ | ND^a^ | 0.9693 | NA | 0.9793 | ND^a^ | ND^a^ | 0.944 | ND^a^ |
| **GD3** |  |  |  |  |  |  |  |  |  |
| Bmax (OD_405nm_) | ND^a^ | ND^a^ | 3.45 | ND^a^ | 3.291 | ND^a^ | ND^a^ | ND^a^ | ND^a^ |
| K_D_ (M) | ND^a^ | ND^a^ | 8.18E-09 | ND^a^ | 7.88E-09 | ND^a^ | ND^a^ | ND^a^ | ND^a^ |
| R squared | ND^a^ | ND^a^ | 0.9922 | ND^a^ | 0.9906 | ND^a^ | ND^a^ | ND^a^ | ND^a^ |
| Mean K_D_±SD (M) for 2 assays | ND^a^ | ND^a^ | 2.36±2.19E-08 | ND^a^ | 2.82±2.68E-08 | ND^a^ | ND^a^ | ND^a^ | ND^a^ |
| **dGD3** |  |  |  |  |  |  |  |  |  |
| Bmax (OD_405nm_) | ND^a^ | ND^a^ | 3.256 | ND^a^ | 3.186 | ND^a^ | ND^a^ | ND^a^ | ND^a^ |
| K_D_ (M) | ND^a^ | ND^a^ | 7.82E-09 | ND^a^ | 3.02E-09 | ND^a^ | ND^a^ | ND^a^ | ND^a^ |
| R squared | ND^a^ | ND^a^ | 0.9904 | ND^a^ | 0.9891 | ND^a^ | ND^a^ | ND^a^ | ND^a^ |
| Mean K_D_±SD (M) for 2 assays | ND^a^ | ND^a^ | 2.5±2.4E-08 | ND^a^ | 3.4±0.041E-9 | ND^a^ | ND^a^ | ND^a^ | ND^a^ |

ND^a^, not determined (no measurable activity or insufficient data over the range of concentrations tested to be able to determine binding data).

**TABLE S2a. Waters bridging Fab to dPSA**

| **Water** | **Fab chain** | **Residue** | **Atom** | **Distance 1 (Å)** | **dPSA residue** | **Atom** | **Distance 2 (Å)** |
| --- | --- | --- | --- | --- | --- | --- | --- |
| S 13 | B | I 96 | O | 2.7 | Y8W 1 | O10 | 2.8 |
| S 65 | A | R 103 | N | 2.9 | 18D 2 | O1 | 2.8 |
| S 68 | A | N 56 | ND2 | 3.0 | Y98 3 | O1A | 2.7 |
| S 68 | A | N 53 | ND2 | 3.3 | Y98 3 | O1A | 2.7 |
| S 95 | A | K 54 | N | 3.2 | Y98 3 | O9 | 3.2 |
| S 95 | A | K 54 | N | 3.2 | Y98 4 | O7 | 3.1 |
| S 95 | A | A 55 | N | 2.8 | Y98 3 | O9 | 3.2 |
| S 95 | A | A 55 | N | 2.8 | Y98 4 | O7 | 3.1 |

**TABLE S2b Intramolecular contacts in dPSA**

| **dPSA Residue** | **Atom** | **dPSA residue** | **Atom** | **Distance (Å)** |
| --- | --- | --- | --- | --- |
| Y8W 1 | O7 | 18D 2 | O7 | 3.1 |
| 18D 2 | O4 | 18D 2 | O9 | 2.7 |
| Y98 3 | O9 | Y98 4 | O6 | 3.1 |
| Y98 4 | O8 | Y98 4 | O1 | 3.1 |
| Y98 4 | N5 | Y98 4 | O7 | 3.3 |

**TABLE S2c Waters bridging dPSA to dPSA**

| **Water** | **dPSA residue 1** | **Atom** | **Distance 1 (Å)** | **dPSA residue** | **Atom** | **Distance 2 (Å)** |
| --- | --- | --- | --- | --- | --- | --- |
| S68 | 18D 2 | O7 | 2.9 | Y98 3 | O1A | 2.7 |
| S94 | Y8W 1 | O10 | 2.8 | 18D 2 | O5 | 2.6 |
| S95 | Y98 3 | O9 | 3.2 | Y98 4 | O6 | 3.1 |

**TABLE S3d Glycerol contacts**

| **Residue** | **Atom** | **Other chain** | **Residue** | **Atom** | **Distance (Å)** |
| --- | --- | --- | --- | --- | --- |
| GOL 308 | O1 | S | S 82 (H_2_O) | O | 2.3 |
| GOL 308 | O2 | S | S 13 (H_2_O) | O* | 2.8 |
| GOL 308 | O2 | dPSA | Y8W 1 | O8 | 3.0 |
| GOL 308 | O3 | B (light) | I96 | O | 3.0 |
| GOL 308 | O3 | B | H98 | N | 3.4 |

*S13 H_2_O bridges Fab chain B I96 O (2.7Å) to dPSA Y8W 1 O10 (2.8Å)

**FIGURE S1.** Sequence comparison between the mouse and humanized huSEAM 3. Residues that make polar contacts with the oligosaccharide are highlighted.

moSEAM 3 LC DIVMTQSPLTLSVTIGQPASISCKSSQSLLHSNGKTYLNWLLQRPGQSPKLLIYLVSKLE

huSEAM 3 LC DIVMTQTPLSLSVTPGQPASISCKSSQSLLHSNGKTYLNWYLQKPGQSPQLLIYLVSKLE

******:**:**** ************************* **:*****:**********

moSEAM 3 LC SGVPDRFSGSGSGTDFTLKISRVEAEDLGLYYCLQIIHFPHTFGAGTKLELKRA

huSEAM 3 LC SGVPDRFSGSGSGTDFTLKISRVEAEDVGVYYCLQIIHFPHTFGQGTKVEIKRT

***************************:*:************** ***:*:**:

moSEAM 3 HC EVKLQESGGGLVQPGDSLSLSCAASGFTFTPYYMGWVRQPPGKALEWLGYIRNKANGYTT

huSEAM 3 HC EVQLVESGGGLVQPGDSLRLSCAASGFTFTPYYMGWVRQAPGKGLEWVGYIRNKANGYTT

**:* ************* ********************.***.***:************

moSEAM 3 HC EYSASVKGRFTISRDNSQSILYLQMNALRAEDSATYYCARYARGTVDSWGQGTTLTVS

huSEAM 3 HC EYSASVKGRFTISRDNSQSSLYLQMNSLKTEDTAVYYCARYARGTVDSWGQGTLVTVS

******************* ******:*::**:*.****************** :***

**FIGURE. S2** Siglec binding to polySia-ADH and dPSA-ADH using BSA a blocking buffer. The ELISA was performed as described in the EXPERIMENTAL PROCEDURES except that the blocking buffer contained 1% (weight/volume) BSA instead of cold-water fish skin gelatin.


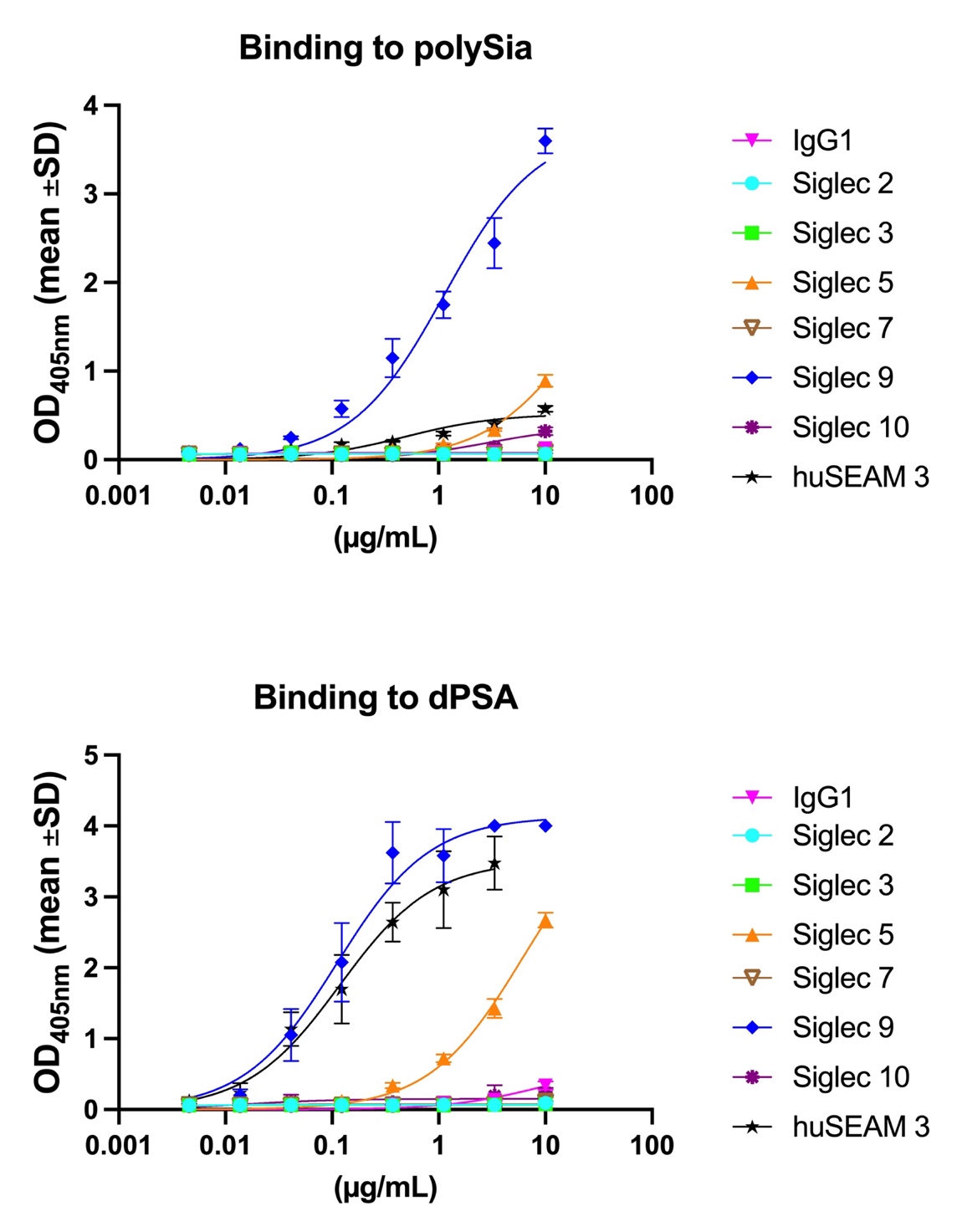


**FIGURE S3**. Superposition of CDRs and dPSA from two independent copies in the asymmetric unit. The heavy chains A and C are shown in cyan and magenta, respectively, the light chains B, D are shown in green and tan, respectively, and the ligand E and F chains are shown in orange and yellow, respectively. The rms deviation for backbone atoms of the Fab is 0.44Å


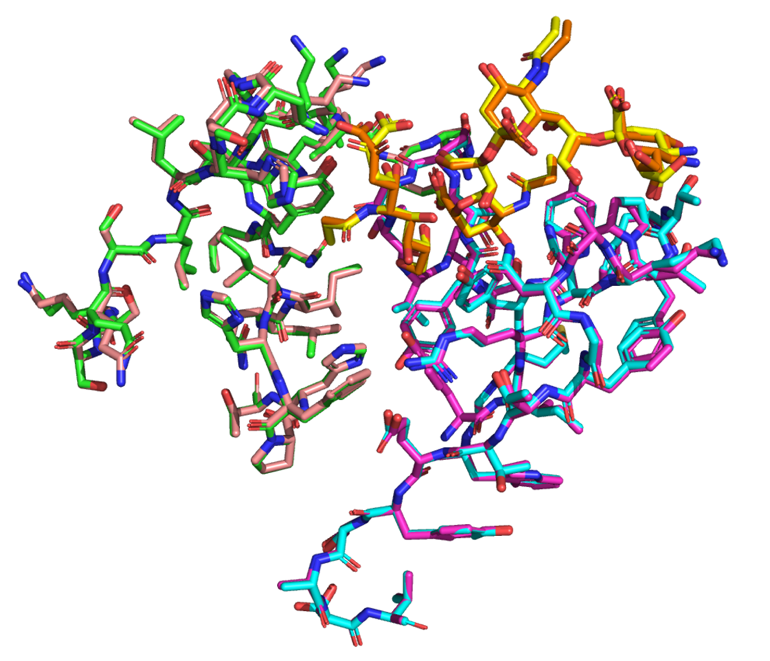


**SEAM 3 binding to a short (Dp<10) dPSA-ADH-biotin antigen by BiaCore surface plasmon resonance.**

SEAM 3 binding to an N-acetyl oligosaccharide dPSA-ADH-biotin antigen adsorbed to BioCore Sensor Chip SA at a concentration of 2µg/mL. SEAM 3 at concentrations of 1 µM, 3.13 µM, 10 µM, 31.62 µM, and 100µM were passed over the chip at a rate of 30 µL/minute. The chip was regenerated between each run with 50 mM sodium hydroxide, 1 M NaCl. The assay was run three times.

Data analysis:

t_c_ 3.467x10^7^,RU•M^-1^•s^-2/3^•m^-1^ k_e_=1.077x10^8^

Chi^2^=12.3 RU^2^, U-value=5

k_a_=2.47x10^6^ (1/M•s)

k_d_=0.01835 1/s

K_D_=7.4x10^-9^ M

**Fab/scFv/Antibody Fragment Complexes with Sialic Acid-Containing Glycans in the PDB**

**1. Anti-Ganglioside Antibodies (sialic acid as antigen)**

These are true cases where the antibody CDRs directly contact the sialic acid:

| **PDB ID** | **Fragment** | **Antibody** | **Antigen** | **Notes** |
| --- | --- | --- | --- | --- |
| **4TUJ** | Fab | 14G2a | GD2 (disialoganglioside) | Reveals that GD2 recognition by 14G2a is based primarily on an extended network of direct and water-mediated hydrogen bonds; comparison with apo Fab reveals an induced-fit binding mechanism. [RCSB PDB](https://www.rcsb.org/structure/4TUJ) |
| **6S2I** | scFv | 14F7 | NeuGc GM3 trisaccharide | 2.3 Å structure of the 14F7 scFv in complex with the NeuGc GM3 trisaccharide; a centrally placed water molecule mediates the specificity of 14F7 for NeuGc over NeuAc GM3. [RCSB PDB](https://www.rcsb.org/structure/6S2I) |
| **3IU4** | Fab | chP3 | NeuGc-GM3 (modelled) | Crystal structure of the chP3 Fab; the modelled complex shows the negatively charged NeuGc is buried in a pocket flanked by two arginine residues, VH Arg31 and VH Arg100A. [RCSB PDB](https://www.rcsb.org/structure/3IU4) Note: the glycan complex is modelled, not co-crystallized. |
| **6UG7 / 6UG8 / 6UG9** | Fab | ch28/11 | SSEA-4 glycan (sialylated) | Five X-ray structures of ch28/11 Fab complexes with the SSEA-4 glycan headgroup at 1.5–2.7 Å resolution; the terminal sialic acid plays a dominant role in dictating the exquisite specificity of ch28/11, and the glycan adopts a horseshoe-shaped conformation in a deep groove. [RCSB PDB](https://www.rcsb.org/structure/6UG9) Three crystal forms (monoclinic, hexagonal, tetragonal). |

**2. Anti-Polysialic Acid Antibodies**

| **PDB ID** | **Fragment** | **Antibody** | **Antigen** | **Notes** |
| --- | --- | --- | --- | --- |
| **3WBD** | scFv | mAb735 | Octasialic acid (α2-8-linked) | 1.8 Å structure; two scFv735 molecules associate with one octasialic acid, with all CDRs except L3 interacting with three consecutive sialic acid residues; 11 ordered water molecules bridge the antibody–ligand gap. [RCSB PDB](https://www.rcsb.org/structure/3WBD) |
| **1PLG** | Fab | mAb735 | Apo (but see 3WBD) | Free Fab; the scFv complex (3WBD) is the actual sialic acid complex. |
| **8FPA** | Fab | huSEAM 3 | dPSA tetramer | 1.83Å structure; four sialic acid residues, three with N-propionyl and one de-N-acetyl at the non-reducing end directly contact four CDRs (H1, H2, H3, L2). [http://www.rcsb.org/structure/8fpa](RCSB%20PDB) |

**3. Anti-Glycopeptide Antibodies (sialic acid on O-glycan)**

| **PDB ID** | **Fragment** | **Antibody** | **Antigen** | **Notes** |
| --- | --- | --- | --- | --- |
| **7C94** | Fab | LpMab-3 | Podoplanin glycopeptide with disialyl-core-1 O-glycan | 2.8 Å structure; two sialic acid residues of the disialyl-core-1 O-glycan (NeuAcα2-3Galβ1-3[NeuAcα2-6]GalNAc) directly contact four CDRs (H1, H2, H3, L3). [RCSB PDB](https://www.rcsb.org/structure/7C94) |

**4. Anti-Viral Hemagglutinin Antibodies (sialic acid binding site mimicry)**

These are Fab–HA complexes where the antibody mimics or blocks the sialic acid binding site, but **the sialic acid itself is generally not present** in the co-crystal:

- **3VFG** (3F8 anti-GD2 Fab — apo, GD2 docked)
- **4HF5, 4HG4, 4HFU** (anti-H2 HA Fabs — contact the sialic acid pocket on HA, but sialic acid not in crystal)
- **5UGY** (CH65 Fab–H1 HA — CDR3 mimics sialic acid binding)

These are important structurally but do **not** contain a sialic acid-containing glycan in the crystal.
